# Translating cancer genomics into precision medicine with artificial intelligence: applications, challenges and future perspectives

**DOI:** 10.1007/s00439-019-01970-5

**Published:** 2019-01-22

**Authors:** Jia Xu, Pengwei Yang, Shang Xue, Bhuvan Sharma, Marta Sanchez-Martin, Fang Wang, Kirk A. Beaty, Elinor Dehan, Baiju Parikh

**Affiliations:** IBM Watson Health, Cambridge, MA USA

## Abstract

In the field of cancer genomics, the broad availability of genetic information offered by next-generation sequencing technologies and rapid growth in biomedical publication has led to the advent of the big-data era. Integration of artificial intelligence (AI) approaches such as machine learning, deep learning, and natural language processing (NLP) to tackle the challenges of scalability and high dimensionality of data and to transform big data into clinically actionable knowledge is expanding and becoming the foundation of precision medicine. In this paper, we review the current status and future directions of AI application in cancer genomics within the context of workflows to integrate genomic analysis for precision cancer care. The existing solutions of AI and their limitations in cancer genetic testing and diagnostics such as variant calling and interpretation are critically analyzed. Publicly available tools or algorithms for key NLP technologies in the literature mining for evidence-based clinical recommendations are reviewed and compared. In addition, the present paper highlights the challenges to AI adoption in digital healthcare with regard to data requirements, algorithmic transparency, reproducibility, and real-world assessment, and discusses the importance of preparing patients and physicians for modern digitized healthcare. We believe that AI will remain the main driver to healthcare transformation toward precision medicine, yet the unprecedented challenges posed should be addressed to ensure safety and beneficial impact to healthcare.

## Background

Is artificial intelligence (AI) going to take over the world as pictured in the sci-fi movies? It has famously beaten the best-performing human beings in competitions such as Jeopardy, AlphaGo, etc., and is now crawling into our daily life without notice. Autonomous vehicles, smart homes, chat bots, individualized marketing, fraud detection, and high-frequency automated trading are some examples of AI empowering humans to live in a more efficient and personalized way. AI augments and is complement to, not a replacement for human intelligence and intuition, where its goal is to help humans become faster and smarter in certain tasks.

Healthcare, an industry that is long governed by medical professionals, is also benefitting from AI. Progress in artificial intelligence and machine learning, along with the accessibility of cloud scaling for big data storage, and integration of health records have expanded the potential for personalized medicine (Syrjala 2018). Information can be automatically extracted and summarized from electronic medical records or from manually written doctor notes by natural language processing (NLP) (Bedi et al. 2015; Chang et al. 2016; Garvin et al. 2018; Meystre and Haug 2006; Miotto et al. 2016; Osborne et al. 2016). Through application of deep learning on medical imaging data, AI has outperformed expert pathologists and dermatologists in diagnosing metastatic breast cancer, melanoma, and several eye diseases (De Fauw et al. 2018; Ehteshami Bejnordi et al. 2017; Haenssle et al. 2018). AI also contributes to innovations in liquid biopsies and pharmacogenomics, which will revolutionize cancer screening and monitoring, and improve the prediction of adverse events and patient outcomes (Cohen et al. 2018; Low et al. 2018). Moreover, AI applications are already playing an important role in fields like gene-editing CRISPR and drug discovery (Abadi et al. 2017; Yu et al. 2017). AI-powered services, such as monitoring health status and suggesting actions to improve well-being through the use of mobile devices and the internet of things (IoT), are entering the market.

In the field of cancer genomics, the availability of multi-omics data, genotype–phenotype data through genome-wide association studies (GWAS), and literature mining has fostered the development of advanced AI techniques and solutions, which allow medical professionals to deliver personalized care through precision medicine (Li et al. 2018; Szymczak et al. 2009; Telenti et al. 2018). Precision medicine is an emerging approach for disease prevention and treatment based on the specific genetics, environment, and lifestyle choices of an individual patient. AI systems are capable of identifying individual drug-response variability (Kalinin et al. 2018; Lin et al. 2018), making recommendations based on patterns learned from vast amounts of public and proprietary data sources, and can help extend the frontier of personalized medicine and specifically of cancer genomics. In this review, we will focus on the existing solutions and applications of AI in the field of cancer genomics and how they are shaping the precision oncology field.

## What is artificial intelligence

AI is the combination of theories, algorithms, and computing frameworks, facilitating various tasks that require human intelligence such as reasoning, decision-making, speech recognition, language understanding, and visual perception. It is one term that encompasses numerous methods such as logic (rule-based), machine learning (ML), deep learning, NLP, and computer vision. AI can help to significantly speed up the process of analyzing vast amounts of data, leverage patterns in the data, and result in faster and better advised decision-making. Sophisticated predictive models are built using algorithms that mine the patterns from data and predict outcomes. As the availability of data in a domain increases, so does the adoption and utilization of such disruptive technologies. With the advent of Big Data and the ever-increasing storage and computing power, the challenge has shifted from collecting data to turning it into meaningful and actionable insights.

## How next-generation sequencing is changing the landscape of cancer genomics

Next-generation sequencing (NGS) is being applied broadly as a valuable method for gaining insights into the genomic profile of a tumor. The ability to simultaneously sequence millions of DNA fragments in a single sample to detect a wide range of aberrations provides a comprehensive profile of the tumor. Due to comprehensive detection of aberrations, combined with improvements in reliability, sequencing chemistry, pipeline analysis, data interpretation, and cost, the adoption of NGS for clinical purposes has grown tremendously (Pennell et al. 2018). Cancer panels are designed specifically to detect clinically relevant somatic mutations with high confidence. Germline mutations in cancer-predisposing genes such as BRCA1/2 are also detected to assess cancer risk. In 2017, the FDA approved several NGS-based panels related to oncology: Oncomine Dx Target Test, Praxis Extended RAS Panel, MSK-IMPACT, and FoundationOne CDx. Recent FDA approval of NTRK gene fusions for tumor-agnostic indications also expands the clinical utilization of NGS (Larotrectinib FDA approval).

Liquid biopsy holds great promise due to its non-invasive nature. Multiple studies have demonstrated the application of liquid biopsy for cancer diagnosis, prognosis, and drug-response monitoring (Palmirotta et al. 2018). Cell-free DNA (cfDNA) released by dying tumor cells, cell-derived vesicles termed exosomes, and circulating tumor cells (CTCs), which shed from the tumor and enter the vasculature system, are often used as a source for tumor DNA. Importantly, a variety of research groups have shown that NGS-sequencing protocols can be modified to achieve sensitivity levels comparable to the standard sequencing procedures (Aravanis et al. 2017), but its implementation in clinical practice is pending confirmation via clinical trials.

The Cancer Genome Atlas (TCGA) project highlights how NGS screens can facilitate the discovery of novel oncogenic mechanisms and patient stratification. The data have been used to elucidate functionally relevant oncogenic mechanisms across multiple tumor types (Cancer Genome Atlas Research Network et al. 2013; Sanchez-Vega et al. 2018; Cava et al. 2018). In a recent study, the regulatory role of F-box/WD repeat-containing protein 7 (Fbw7) in cancer cell oxidative metabolism is discovered (Davis et al. 2018) using ML algorithms. Molecular subtypes discovered in the pan-cancer studies also help to personalize the treatment and improve patient’s survival outcome (Cancer Genome Atlas Network 2012; Curtis et al. 2012; Vaske et al. 2010).

Finally, NGS supports the discovery of novel biomarkers such as mutation signatures and tumor mutational burden (TMB). Statistical analyses are performed, and patterns are discovered through millions of mutations detected by NGS. TMB has been shown to be an effective biomarker for predicting the response to immuno-therapy—an innovative area of research that can use the body’s own immune system to fight cancer (Steuer and Ramalingam 2018). For all of these reasons, NGS has proven to be a powerful tool in clinical oncology. However, important challenges remain in cancer genomics and precision medicine fields such as efficiently leveraging the vast amount of genomic data available and making relevant treatment recommendations to clinicians.

## Challenges in cancer genomics data interpretation

Next-generation sequencing has revolutionized medical research and enabled multi-layer studies that integrate genomic data of high dimensionality such as DNA-seq, RNA-seq, and other multi-omics data such as proteome, epigenome, and microbiome. The integrative analysis of multi-omics data provides a more comprehensive view of biological processes leading to a better understanding of these systems compared to single-layer analysis (Chari et al. 2010; Wang et al. 2014).

However, there are several challenges to the translation of multi-omics data into clinically actionable biomarkers. First, combing data profiles at various levels would result in high dimensionality with large number of covariates. Data sparsity from high dimensionality combined with high heterogeneity from diverse types of data imposes a significant difficulty in integrative analyses. Many dimension reduction techniques such as multiple co-inertia analysis and multiple factor analysis have been developed to facilitate downstream joint analyses by mapping the data to lower dimensional space without a significant loss of information and transforming observations across data sets (Meng et al. 2016). Various integrating frameworks especially network-based approaches which use graphical algorithms to capture molecular network interactions and multi-level Bayesian models which impose realistic assumptions for parameter estimation through a prior-posterior Bayesian structure have been commonly applied in advanced strategies for multi-omics data analysis (Bersanelli et al. 2016).

Second, better standards for data generation and reporting are needed to facilitate data integration and to reduce bias (Ibrahim et al. 2016; Li et al. 2017). Sample acquisition and preparation procedures need to be well regulated for data generation and sequencing platform, and computational pipelines need to be carefully calibrated and validated. For instance, for NGS data, reference material (CLSI QMS01-A 2018; CLSI MM01A3E 2018; NIST 2018) whose properties are sufficiently homogeneous and well established to be used for the calibration of sequencing system is needed. The Centers for Disease Control (CDC) and Prevention’s Genetic Testing Reference Material Coordination Program (GeT-RM) is engaging to generate renewable and publicly available characterized gDNA reference materials that can be used for clinical NGS testing. Other than reference materials, laboratory practice guidelines were published by CDC’s Nex-StoCT II working group (Gargis et al. 2015). However, since hardware and software often get updated frequently and NGS analysis often encompass complex multi-step processes, further guidance for quality control criteria is needed, especially when sharing data among different laboratories. Those standards will help different laboratories to validate procedures, assess the quality of sequencing, evaluate performance of new platforms, and compare or share results among them.

Last, but not least, well-designed studies with causal inference are needed to filter out biomarkers that have strong correlative effects but no real causative effects in tumorigenesis (Ibrahim et al. 2016; MacArthur et al. 2014). Multiple classes of evidence may contribute to the pathogenic inference, including genetic, informatic, and experimental data. On the genetic level, the pathogenic variants could be significantly enriched in cases compared to controls and/or the variant is co-inherited with disease status within affected families. On the informatic level, the pathogenic variants could be found at the location predicted to cause functional disruption (for example, protein-binding region). And on the experimental level, the pathogenic variants could significantly alter levels, splicing, or normal biochemical function of the product of the affected genes. This can be shown either in patient cells or well validated with in vitro or in vivo models such as introduction of the variant or an engineered gene product carrying the variant into cell lines or animal models results in phenotype consistent with the disease. Finally, the cellular phenotype in patient-derived cells, model organisms, or engineered equivalents can be rescued by addition of wild-type gene product or specific knockdown of the variant allele (MacArthur et al. 2014). Careful attention should be drawn on these aspects in regard to evaluating pathogenicity of new discovered biomarkers from omics data.

The advancement of ML technologies is bound to impact the interpretation of genomic sequencing data, which has traditionally relied on manual curation by experts in the field. These curation efforts rely on protein structure, functional studies and more recently, on “in silico” models that predict the functional impact of genetic alteration such as SIFT, PANTHER-PSEP, PolyPhen2, and others (Tang and Thomas 2016). Genomic databases such as ClinVar or COSMIC have proliferated as means of concisely compiling a collection of classified genetic variants. They provide the evidence supporting the classification of a variant as being pathogenic, benign or of unknown significance (VUS).

Two key limitations of manually curating and interpreting the results from genomics data are scalability and reproducibility. These challenges continue to grow as more genomic data become available. The number of curation experts or variant scientists and the amount of time that they can dedicate daily to this task is limited. Different variant scientists among companies, research groups, and hospitals can introduce bias due to subjectivity in curation criteria, adherence to Standardized Operating Procedures and training. To address these limitations, organizations are working to build and standardize multi-step protocols for variant classification such as the American College of Medical Genetics and Genomics and the Association for Molecular Pathology (ACMG-AMP), who, in 2015, published a series of guidelines for the interpretation of germline genetic variants for genes causative of hereditary human disorders (Richards et al. 2015). These guidelines have been adopted, refined, and tested in multiple institutions for several genetic diseases including cancer, Marfan Syndrome, and diabetes among others (Amendola et al. 2016; Muino-Mosquera et al. 2018; Richards et al. 2015; Santana et al. 2017; Sukhai et al. 2016). Similarly, the International Society for Gastrointestinal Hereditary Tumors (InSiGHT) has developed a standardized classification scheme for variants occurring in genes associated with hereditary gastrointestinal tumors such as Lynch Syndrome (Thompson et al. 2014). More recently, ACMG and AMP in collaboration with the American Society of Clinical Oncology, and College of American Pathologists have published guidelines for the classification, annotation, interpretation, and reporting for somatic sequence variants in cancer (Li et al. 2017). Yet, the ability to scale NGS variant interpretation and to maintain strict quality control remains limited.

## Precision medicine and AI

**Fig. 1:**
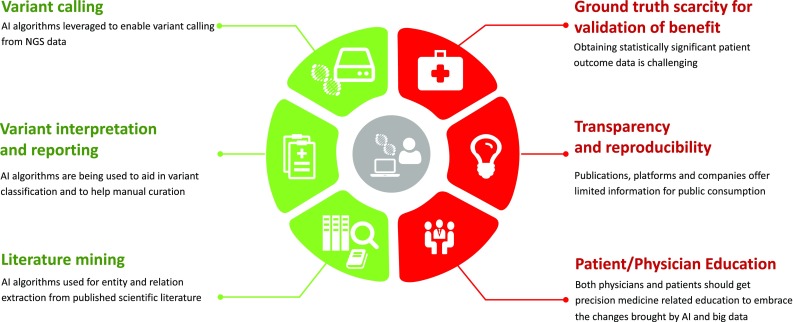
Topics discussed in the review paper. This figure demonstrates that several key topics discussed in the paper with the green icons representing benefits or improvements and red icons representing challenges or caveats

Precision medicine or personalized medicine tackles diseases by tailoring treatment based on genomic, lifestyle, and environmental characteristics of each patient. With precision medicine and the advancement of NGS, genomic profiles of patients have been increasingly used for risk prediction, disease diagnosis, and development of targeted therapies. Gene expression is an important part of the patients’ genomic profiles, and interestingly, ML classification methods applied to gene expression data are not new. Historically, comprehensive gene expression analysis was done with microarrays and now with RNA-seq. Expression data are analyzed to identify the significant genes in the upregulated or downregulated pathways (Lyu and Haque 2018; Hwang et al. 2002), and are also trained to predict the cancer subtypes and prognosis when outcome data or diagnosis information is available (Bartsch et al. 2016; Pepke and Ver Steeg 2017). Multiple review papers have already covered different ML applications on gene expression data (Molla et al. 2004; Sajda 2006; Kourou et al. 2014; Libbrecht 2015; Bashiri et al. 2017; Noor and Narwal 2017). In our review, we, however, will focus on AI applications related to NGS and cancer genomics testing (Fig. 1).

### Variant calling

Variant calling is the process to identify variants in NGS data. Raw sequencing reads are first aligned to the reference genome and then run through multiple quality improvement steps to prepare for the variant calling (e.g., quality evaluation, recalibration, indel realignment, and identifying duplicates). Randomness of DNA molecule selection at the enrichment step, platform-dependent systematic errors, sequencing errors, and alignment errors constitute the main challenges of this step.

Out-of-box usage of most variant callers is not ideal, especially in a clinical setting. Often, users need to heuristically tune parameters and apply multiple customized filters to remove false positives before an acceptable accuracy is achieved. This is a time-consuming effort that requires expertise to fine tune quality scores and attributes within contexts of sequencing, amplicon, alignment, and genomics.

Different groups are now leveraging ML algorithms and training on those underlying quality features such as sequencing and alignment quality to improve the performance of the variant calling, especially in sub-optimal scenarios (Ding et al. 2012; Hao et al. 2017; Hill et al. 2018; Spinella et al. 2016). Tumor ploidy and purity are two major factors that contribute to cancer complexity. Sub-clonal variants (present only in a few cells) are difficult to detect, because their representation in the sequencing library is low. This can result in variability across analysis methods, thresholds, and quality scores which may not be flexible enough to detect sub-clonal variants. Instead of setting up static rules, ML methods are able to adjust the thresholds dynamically based on the patterns. Variants with very low allele frequencies can still be reported if the sequencing depth and other quality metrics outperform and pass the overall confidence threshold. For instance, a convolutional neural network (CNN) model of which the algorithms are often used in image recognition achieved F1 score of 0.96, and was able to reach variants with allele frequency as low as 0.0001 (Hill et al. 2018). F1 score is an accuracy measure that takes into account both precision and recall. In another instance, a Random Forest-based ML approach (Cerebro) applied to NGS data showed improved accuracy, as measured by F1 score, in the identification of tumor mutations when compared to the existing variant calling programs such as MuTect1, MuTect2, SomaticSniper, Strelka, VarDict, and VarScan2. While their recall values are fairly similar, Cerebro showed increased precision values comparing to the other methods (Wood et al. 2018). Similar successes of ML have been described in copy-number variation (CNV) analyses (Antaki et al. 2018; Onsongo et al. 2016).

Besides standard variant detection paradigms, Google’s DeepVariant transforms a variant calling problem into an image recognition problem by converting a BAM file into images similar to genome browser snapshots and calls the variants based on likelihoods, using the Inception Tensor Flow framework which was originally developed for image classification (Going Deeper with Convolutions 2014). Another recent study successfully applied ML on sequencing data from multiple regions of a tumor to identify and learn growth patterns as accurate predictors for tumor progression (Caravagna et al. 2018).

### Variant interpretation and reporting

Variant annotation and classification are the basis of genetic diagnostics and are crucial to clinical patient care and treatment planning. In vivo or in vitro functional studies are considered the gold standard for determining whether a mutation is benign or disease causing. Several computational methods have been applied for the identification of cancer driver mutations based on non-random distribution of mutations within proteins (Porta-Pardo et al. 2017). In silico prediction tools like PolyPhen and SIFT are widely used to assist the manual curation but have not established themselves as the determining factors in the clinical setting (Adzhubei et al. 2010; Vaser et al. 2016). Many research groups are training ML models on features encoding secondary structures, intrinsic disorders, DNA-binding, phosphorylation, conservation, predicted structure, and homolog counts to further improve the accuracy of variant classification, to incorporate high-dimensional data sets, and to unify the variant interpretation among laboratories. Some notable examples are deep neural networks (Bromberg et al. 2008; Ferrer-Costa et al. 2005; Qi et al. 2018; Quang et al. 2015), decision tree (Dobson et al. 2006; Krishnan and Westhead 2003), random forrest (Bao and Cui 2005; Carter et al. 2009; Kaminker et al. 2007; Li et al. 2009a; Wainreb et al. 2010), and support vector machine (Calabrese et al. 2009; Capriotti et al. 2006, 2008; Karchin et al. 2005; Yue and Moult 2006).

It is also imperative to evaluate and validate prediction tools. The critical assessment of genome interpretation (CAGI) has carried out prediction challenges accompanied with experimentally confirmed validated results throughout the years. The shared data and assessment publications are invaluable sources to set the standards for evaluation on the performance of any prediction tool. For example, the proprietary classification for BRCA mutations carried by Myriad Genetics has been considered an established assessment to evaluate pathogenicity in functional studies. Using ensemble learning methods on multimodal data sets, Pejaver et al. have developed missense variant pathogenicity predictors with high accuracy of predictions on BRCA missense variants classified by Myriad Genetics. Unfortunately, their evaluation is inconclusive due to small sample size, and only a small number of mutations were evaluated in this study (Pejaver et al. 2017). Saturation genome editing has been used in a recent study to assay all possible single-nucleotide variants in functionally critical domains of BRCA1 for variant functional classification and could be a viable strategy to overcome the challenge of lacking variant functional data, especially for those rare and low-frequency variants and to enable approaches with systematically derived measurements for functional analysis (Findlay et al. 2018).

Cancer biologists and molecular pathologists are trained to classify cancer sequence variants for their pathogenicity and clinical relevance. This is a complex process which is difficult to compile into a set of rules comprehensive enough to cover all scenarios. To what degree can ML algorithms learn the complex clinical decisions made by individual pathologists and classify the variants automatically? Massachusetts General Hospital (MGH) did the experiment and got very promising results. They selected ~ 500 features, built multiple ML models on ~ 20,000 clinical sign-out variants reported by board-certified molecular pathologists and then compared the prediction results to find the best model (Zomnir et al. 2018). The logistic regression model demonstrated the best performance with only 1% false negativity and 2% false positivity, which is comparable to human decisions.

## Literature mining

**Fig. 2:**
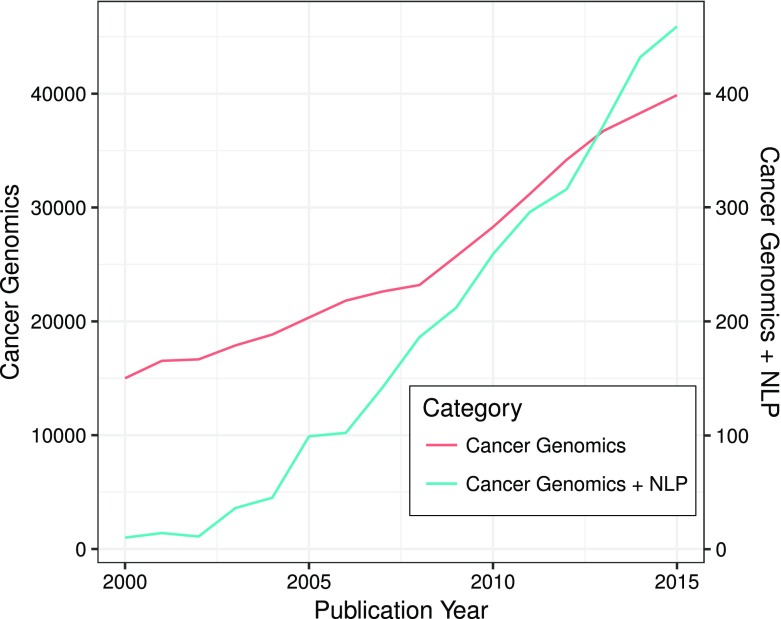
Publication number plotted against publication year. In this figure, two *y*-axes have been plotted. One *y*-axis represents the number for papers related to “Cancer Genomics”. The other *y*-axis represents the number for papers related to “Cancer Genomics + NLP”. The *x*-axis represents the publication year

Owing to the open-access policies of many journals and the steady growth of scientific publications (Fig. 2), there is widespread availability of the published literature. PubMed currently comprises of over 28 million citations from Medline, life science journals, and online books (PubMed). The number of publications each year, as indexed in PubMed, has exceeded 1 million since 2011. This volume and veracity in publications indicate multiple hypotheses are being tested at the same time, which makes it harder for researchers to stay up to date in their field in the absence of some automated assists. It, therefore, impacts their ability to generate meaningful and coherent conclusions in a timely manner which are required for evidence-based recommendations in the context of precision medicine (Harmston et al. 2010). Applications and use of NLP-based solutions reduce the time and effort required for information retrieval (IR) and speed up curation, and provide novel opportunities for hypothesis generation based on the published literature (Caporaso et al. 2008; Extance 2018). In cancer genomics, publications per year can easily run into tens of thousands—far more than a researcher can keep up with—and this growth in publication has resulted in rapid growth of application of text mining and NLP techniques (Fig. 2). Biomedical named entity recognition (Bio-NER) and relationship extraction are two key NLP processes used in evidence extraction. The publicly available tools are reviewed and compared here (Table 1).

**Table 1:** Survey list of selected tools or algorithms for Bio-NER and relationship extraction in genomics

Category	Paper	Tool_Name	Extraction_Target	Algorithm_Model	License_Availability	Evaluation_Corpus
Name entity	Wei et al. (2013)	tmVar	Mutation	CRF (conditional random field) + rule-based	NCBI; accessible by RESTful API	PubMed abstract
	Doughty et al. (2011)	EMU	Mutation	Rule-based approach	Freely available	Inhouse corpus
	Caporaso et al. (2007)	MutationFinder	Mutation	Rule-based approach	source code available in JAVA, PYTHON, PERL	Inhouse corpus
	Thomas et al. (2016)	SETH	Mutation	Extended Backus–Naur Form (EBNF) grammar	Freely available	A series of pervious available corpus
	Settles (2005)	ABNER	Genes, protein	CRF	Open source	Inhouse corpus
	Leaman and Gonzalez (2008)	BANNER	Genes	CRF	Open source	BioCreative 2 GM task
	Wei et al. (2015)	GNormPlus	Genes	CRF + additional information	Open source	BioCreative II GN corpus and Citation GIA test collection
	Rocktaschel et al. (2012)	ChemSpot	Drugs	CRF + dictionary	Freely available	SCAI corpus and IUPAC test corpus
	Leaman et al. (2015)	tmChem	Drugs	CRF	RESTful API	CHEMDNER task
	Lee et al. (2016b)	BEST Biomedical Entity Extractor	Gene, disease, drug and cell line names	Dictionary-based	Freely available	BRONCO
	Leaman et al. (2013)	Dnorm	Disease	Machine learning	RESTful API	NCBI disease corpus
	Leaman and Lu (2016)	TaggerOne	Disease and chemical	Semi-Markov Models	Open source	NCBI disease corpus and BioCreative V Chemical-Disease Relation corpus
Relationship	Pletscher-Frankild et al. (2015)	Diseases	Disease gene	Dictionary and co-occurrence	Freely available	Inhouse corpus
	Mahmood et al. (2016)	DiMeX	Disease mutation	Lexical and semantic patterns + additional information	–	Bio_muta project
	Ravikumar et al. (2015)	MutD	Protein-mutation disease	Dependency parse graph	Plan to release web and RESTAPI	Abstracts from Pubmed articles
	Zou et al. (2017)	IBRel	microRNA gene	Multi-instance learning	Open source	Bagewadi’s corpus
	Burger et al. (2014)	Mturk	Gene mutation	Crowdsourcing	Open source	Inhouse corpus
	Mallory et al. (2016)	DeepDive	Gene interactions	Distant supervision	Open source	Inhouse corpus
	Quirk and Poon (2017)	DISCREX	Drug–gene	Distant supervision	–	Inhouse corpus
	Barbosa-Silva et al. (2011)	PESCADOR	Gene/protein interactions	Co-occurrence	Web application	AIMed corpus
	Bravo et al. (2015)	BeFree	Gene disease, drug disease, and drug-target	Shallow Linguistic Kernel, Dependency Kernel KDEP	Code available upon request	EU-ADR corpus and GAD corpus
	Song et al. (2015)	PKDE4J	Protein–protein interactions, gene–disease, and disease–drug	Dependence parsing-based rules	Publicly available	BioInfer,AIMed (protein–protein interactions); GAD, CoMAGC, Gene–cancer (disease-gene), PolySearch (drug-disease)
	Tsuruoka et al. (2011)	FACTA+	Various binary relationship	Joint CRF learning model	Web application	BioNLP’09 shared task corpus
	Rinaldi et al. (2014)	OntoGene	Various binary relationship	Rule-based approach and maximum entropy	RESTful API	Inhouse corpus
	Poon et al. (2014)	Literome	Various binary relationship	Dependency graph and co-occurrence	Freely available for non-commercial usage	Inhouse corpus
	Liu et al. (2015)	PolySearch2	Various binary relationship	Bag of words + dictionaries	Freely available	Inhouse corpus
	Xu and Wang (2013)	–	Drug gene	Co-occurrence	–	MEDLINE abstracts
	Percha and Altman (2015)	–	Drug gene	Dependence graph generated by Stanford Parser and Ensemble Biclustering for Classification	–	Inhouse corpus
	Singhal et al. (2016)	–	Disease mutation	Decision tree, multi-layer perceptron and Bayesian logistic regression	–	EMU, PubMed_data set generated inhouse
	Muzaffar et al. (2015)	–	Treatment disease	Machine learning	–	Corpus obtained from MEDLINE 2001
	Poon et al. (2015)	–	Pathway interactions	Distant supervision	–	Inhouse corpus
	Miwa et al. (2009)	–	Protein–protein interactions	Combining kernels	–	Aimed, BionInfer, HPRD50, IEPA, LLL corpus
	Yang et al. (2011)	–	Protein–protein interactions	Weighted multiple kernel learning-based approach	–	Aimed, BionInfer, HPRD50, IEPA, LLL corpus
	Bui et al. (2011)	–	Protein–protein interactions	Somatic properties + machine learning	Open source	Aimed, BionInfer, HPRD50, IEPA, LLL corpus
	Tikk et al. (2010)	–	Protein–protein interactions	Kernel-based approach	–	Five publicly available annotated corpora
	Thomas et al. (2011)	–	Drug–drug interaction	Ensemble learning	–	DDI Extraction 2011 challenge
	Bui et al. (2014)	–	Drug–drug interaction	Feature-based machine learning	Open source	2011 and 2013 DDI extraction challenge
	Bundschus et al. (2008)	–	Disease-treatment and genes-disease	CRF	–	GeneRIFs data set and annotated MEDLINE abstracts
	Lee et al. (2018)	–	Mutation-gene-drug	CNN	–	Inhouse corpus
	Peng et al. (2017)	–	n-ary relationship	Graph LSTM	–	Inhouse corpus
	Hakenberg et al. (2012)	–	Various binary relationship	Rule-based approach	–	Inhouse corpus

Tools were evaluated with respect to selected technical criteria including extraction target, algorithm, license, and evaluation corpus, and were grouped into named entity recognition (NER) and relation extraction categories

### Entity extraction

Bio-NER is the foundation of evidence extraction for precision medicine. In cancer genomics, NLP tools have been used for the automated extraction of entities such as gene, genetic variants, treatments, and conditions. Identifying genetic variants is a key step for tumor molecular profiling and downstream gene–protein or gene–disease relationship analysis. Medical providers require the accurate identification and interpretation of genetic variation data to design effective personalized treatment strategies for their patients. Unfortunately, there is no universal standard for how genetic variants are called out and there are multiple ways of presenting the same event in the literature and genomic databases. Variation could be described at multiple description levels such as genomic and protein levels, and mapped against different reference genomes. Their mentions are often written as various natural language phrases besides the standard alphanumeric formats (HGVS format). To consolidate the knowledge on genetic variation from the literature mining and integrate it with curated data in existing resources such as ClinVar and COSMIC, it is essential to both standardize the genetic variations to HGVS nomenclature and normalize them to unique identifiers such as reference SNP ID number (RSIDs).

Current biomedical named entity recognition techniques fall into three major categories: dictionary-based approaches, rule-based approaches, and ML/deep learning approaches (Cohen and Hersh 2005; Li et al. 2009b). Dictionary-based approaches tend to be fast and simple but often miss undefined terms that are not mentioned in the dictionary, while rule-based approaches usually require handcrafted rules that identify terms from text but could be too specialized to adapt to new entity types (Rebholz-Schuhmann et al. 2011). ML approaches generally require the standard annotated training data sets for which the generation process is usually time- and labor-consuming (Krallinger et al. 2011). Recently, several deep learning methods have been applied to biomedical named entity recognition showing large performance gain by better integrating multi-dimensional features and, at the same time, minimizing manual feature generations (Habibi et al. 2017; Wu et al. 2017).

### Relationship extraction

Relationships between recognized entities from the biomedical literature are key to identify the associations of genetic alterations, conditions, and treatments. These can be used as evidence for genetic test reporting by linking genotype-to-phenotype data, such as an association of a specific variant with drug sensitivity or of a variant with predisposition to a specific cancer type. The most intuitive and fastest approach for relation extraction is co-occurrence analysis, which tends to achieve high recall but low precision (Cheng et al. 2008; Doughty et al. 2011; Lee et al. 2016a). A rule-based approach can achieve higher precision (Hakenberg et al. 2012), but defining those rules can be time-consuming and labor-intensive. Many more sophisticated learning methods have been developed over the last decade and they conceptually fall into three categories: the supervised learning approach, the unsupervised or semi-supervised learning approach, and the hybrid learning approach. Within the scope of supervised learning, several papers focused on feature-based approach (Rink et al. 2011; Xu et al. 2012), while several other papers mainly used kernel-based approach (Kim et al. 2015; Ma et al. 2015; Tikk et al. 2013; Yang et al. 2011). Supervised approaches commonly require expensive labeled training data and their feature engineering and kernel selection would be time-consuming. Unsupervised learning approaches, on the other hand, focus on learning inherent structure in data and do not require labeled training data (Alicante et al. 2016; Quan et al. 2014). Unsupervised learning approaches such as association mining can help to identify interesting associations. Since the field of cancer genomics is rapidly evolving, to come up with meaningful evidence-based recommendations, it is important to make sense of vast available data sets and publications. Association mining has been used to identify frequently co-occurring entities to develop meaningful conclusions and recommendations (Alves et al. 2009). Other unsupervised technique such as clustering is used to develop insights into cancer signatures from multi-omics data. Semi-supervised learning such as the distant supervision approach usually utilize weakly labeled data derived from a knowledge base, which has been explored in cancer research (Quirk and Poon 2017). Hybrid approaches usually integrate pattern, rules, domain knowledge, and learning-based methods together to build models (Muzaffar et al. 2015). Deep learning integrates both supervised and unsupervised features by applying multi-layer non-linear functions for analysis and classification. Over recent years, deep learning methods like CNNs (Lee et al. 2018) and recurrent neural networks (RNNs) (Peng et al. 2017) have been applied into the relation extraction field and have led to promising results. Lee et al. demonstrated that CNN can be used for sentence-level relation classification. With features combining word embedding, type embedding, and position embedding, they achieved F1 score of 0.954 and 0.845 for mutation-gene and mutation-drug relationship classification, respectively, without explicitly defining keywords for relation extraction. Peng et al. showed that, by applying graph LSTM for drug–gene–mutation ternary relation extraction, they achieved precision 0.75 (with output probability threshold 0.9) in cross-sentence setting. In addition, graph LSTM outperformed a well-engineered feature-based classifier in extracting genetic pathway interactions using GENIA Event Extraction data set, illustrating its advantage of handling sparse linguistic patterns without intense feature engineering.

## Challenges to AI adoption in healthcare

### Lack of ground truth to validate the benefit

The evaluation of AI accuracy is critical to help gauge how well the system performs in assisting experts, and to make AI less of a black box. In cancer genomics, variant classification, clinical relevance, literature validation, and summarization are traditionally done by human experts. To prove the usefulness of an AI application, it needs to be evaluated in comparison with human experts and not only with the other AI solutions. However, this is rarely done due to the lack of publicly accessible knowledge bases for ground truth data.

Increasingly abundant patient genomic and clinical data generated from various genomic testing platforms are enabling AI solutions to discover novel clinically relevant patient subgroups for better clinical outcome (Kristensen et al. 2014; Kalinin et al. 2018). However, the difficulty of getting a statistically significant patient outcome data is one of the most pressing challenges to achieve an impactful solution. Patient outcome data are personal health information (PHI) that must be protected by the HIPAA guidelines in the US and GDPR in Europe. Given such regulations, sharing such data is not done lightly, as security considerations are vital to preventing sensitive data from being compromised (General Data Protection Regulation 2016; The Health Insurance Portability and Accountability Act of 1996 2014). Furthermore, there is a scarcity of patient outcome data available to be used in training and evaluation of AI systems for guiding the decisions of clinicians and experts in the design of treatment plans.

### Transparency and reproducibility

AI is a hot field and its use has been claimed by many platforms and companies. However, detailed information on AI techniques and models is not clearly presented and there is considerable variability in methodologies from company to company. Based on publicly accessible information, we classify five different levels of transparency with a list of examples (Table 2).

**Table 2:** Major functionalities and transparency for key players in text mining and personalized medicine field

Players	Functionality	Transparency
Blueprint genetics	Offers single gene test, targeted variant testing or whole exome sequencing service along with interpretation	No explicit AI description
Cambridge cancer genomics	Uses blood tests to guide cancer therapy	No explicit AI description
Deep gene	Provides cancer-type classifier based on deep learning and somatic point mutations	Publication is available (Yuan et al. 2016)
Deep genomics	Develops genetic medicines using artificial intelligence technology, with a focus on the preclinical development of oligonucleotide therapies	No detailed explanation but related publication is available (Wainberg et al. 2018)
DeepVariant	Analysis pipeline using a deep neural network to call genetic variants from NGS DNA data	Available in GitHub https://github.com/google/deepvariant
Genomenon	Genomic search engine and database to provide disease-gene-variant relationships from the full text of the scientific literature for gene and variant interpretation	No explicit AI description
Genoox	Fully customized platform for genetic applications including primary, secondary and tertiary analyses	No explicit AI description but related publications available (Stajkovska et al. 2018)
Literome	Automatic curation system to extract genomic knowledge from PubMed articles to facilitate browsing, searching, and reasoning	Publications are available (Poon et al. 2014, 2015)
Perthera	Manage process from tumor testing through Perthera Report to provide cancer patients and physicians with therapeutic options ranked by the probability of outcome	No explicit AI description
Sophia Genetics	Provides NGS data analysis to detect, annotate and pre-classify genomic variants associated to multiple disorder areas	No explicit AI description
Watson for Genomics	Provides in-depth clinical interpretation of the genetic alterations in the sample automatically, enabling clinical decision-making for personalized cancer care	No explicit AI description but related publication is available (Patel et al. 2018)
WuXi NextCODE	Uses genomics to identify the underlying biology and advance the scientific understanding of disease and propel the next generation of transformative therapies	No explicit AI description but related publication is available (Zhang et al. 2018)

For each company, the main functionality and transparency are summarized

Reproducibility of experimental results is the hallmark of science. Therefore, being able to replicate ML results and experiments is paramount. Because ML algorithms typically have lots of tunable components, performance can be affected by the sensitivity to the scale and quality of training data, empirical setting of hyperparameters, and initialization and optimization processes. Many publications fail to disclose simplifying assumptions or implementation details, and thus make it hard to reproduce results. This coupled with the fact that researchers often do not share their source code makes reproducibility a major challenge. Even if all details were shared, reproducibility is not easy to implement, as this requires that we either expect reviewers (a) to very carefully study the code and scripts needed to produce the results or (b) to create a new script based on the description of the algorithm and parameters in the paper. Simply running the scripts and checking whether the tables and graphs of the paper can be reproduced would do little to validate the work. These discussions promote the publication of well-described research methods and protocols, and thus help the advancement and adoption of AI technologies (Hutson 2018). Even after such hurdles are overcome, large-scale deployment of AI solutions in healthcare may happen only when benefits are realized via closely monitored and formally tested assessment in real world.

### Patient/physician education

Digitization of healthcare has provided the access to big-data information and cognitive insights to both caregivers and patients, transforming healthcare and clinical workflows (Mesko et al. 2017). The point-of-care has shifted from the clinic and physician to the patient. The old paradigm of paternalistic physician–patient relationship has been transformed into an equal-level partnership with shared medical decision-making. Experience-based medicine has evolved into evidence-based and patient-centered approaches. Both physicians and patients need to be prepared for this revolutionary role of AI in healthcare (Mesko et al. 2017).

Medical professionals must learn how to work alongside data-enabled technology applications and acquire knowledge about how AI works for healthcare delivery. Precision medicine relies on an increasing amount of heterogeneous data of molecular genetics, clinical, and biological parameters for each patient. The total number of parameters for medical decision-making on a single patient could be up to 10,000 by 2020 (Abernethy et al. 2010). It becomes impossible for a physician to bear with all the responsibilities of data management and analysis, not to mention patient communications. Physician burnout has become a pressing health challenge. The application of AI in healthcare aims to advise clinicians with better and faster insights to ultimately improve the lives of patients. By embracing AI, clinical teams could be relieved from repetitive daily work and have more time to focus on the other aspects of patient care (Fogel and Kvedar 2018). For instance, in one study, Watson for Genomics identified genomic alterations with potential clinical impact that were not recognized by the traditional molecular tumor boards in 323 (32%) of patients using an analysis that took only a few minutes (Patel et al. 2018). At MGH, the clinical implementation of an AI-based decision support tool for variant reporting allows molecular pathologists to quickly make decisions and empowers them to explore the underlying reasoning behind them (Zomnir et al. 2018). As we move to an age of AI, medical education must move beyond the foundational biomedical and clinical sciences to knowledge of information platforms and intelligence tools in healthcare and the skills to effectively use them (Wartman and Combs 2018).

On the other hand, capturing data on individual variability in genomic, lifestyle, and clinical factors is at the core of precision medicine, which would empower patients to be more engaged in their health care. With augmented direct access to health and innovative technologies, transparency in healthcare would be improved and may lead to enhanced accountability and productivity. However, at the same time, the risk of patients getting exposed to unreliable or misinterpreted information and turning to non-validated and unregulated technological solutions is increasing (Mesko et al. 2017). To facilitate patient participation in this AI-empowered digital health transformation, medical professionals should provide robust patient education initiatives related to precision medicine, benefits and risks of AI, data sharing, and protection. Healthcare providers need to be sensitive to varying degrees of patient preferences for privacy and properly obtain consent for patient data collection and use. The awareness of patients’ rights and health literacy should be promoted to help patients navigate the modern technology-intensive healthcare system and to become accustomed to shared decision-making. Ethical principles should be developed to help ensure that development and use of AI applications, specifically within healthcare, are accurate, understandable, and beneficial.

## Perspectives: 5 years down the road

We have entered the advent of an era in which AI can help across the medical continuum from research to prognosis, therapy, and post cancer treatment care. AI will remain the main driver to healthcare transformation towards precision medicine. While digital health has become essential for providing best practice in healthcare, it raises some unprecedented challenges (Bibault et al. 2016; Mesko et al. 2017). How this revolutionary role of AI in healthcare translates into an improvement in the actual lives of patients remains to be demonstrated and will be dependent on the availability of patient outcome data. More and more crowd-source challenges will be uniquely designed for problems in cancer genomics with experimentally defined ground truth to objectively and transparently evaluate the accuracy (CAGI 2018; Dream Challenges 2018; Grant Challenges 2018; PrecisionFDA True Challenge 2018). The data sets that will get published following these efforts will help to establish the standards for benchmarking and testing novel algorithms in the cancer community. Data protection, data sharing, and international standardization will be addressed and regulated (Center for Data Innovation). All those unprecedented challenges digital health poses should be addressed to ensure AI safety and beneficial impact to healthcare.
